# Preparing families and institutions to manage anaphylaxis

**DOI:** 10.1186/2045-7022-1-S1-S61

**Published:** 2011-08-12

**Authors:** Angel Mazon

**Affiliations:** 1 Children's Hospital La Fe, Unit of Pediatric Allergy and Pneumology, Valencia, Spain

## 

Most (57-78%) of the recorded anaphylactic reactions in children occur at home and, second, in school or during school activities (5-22%). Children with food allergy are at the highest risk for death, especially if they also suffer from asthma, as most of the deaths attributed to anaphylaxis are due to severe unresponsive bronchospasm. There is a need of education of families and schools on how to prevent and manage anaphylaxis. The preparedness of schools is very variable across Europe, due to various health and education systems. In a national survey of school nurses in UK, more than 80% felt confident in the management of respiratory distress, airway obstruction and anaphylaxis, 77% had adrenaline and albuterol available, and 13% had oxygen. The situation in most countries of Europe is much worse than this, as school nurses are not present in many schools. The education for both families and schools must be directed to provide skills on how to identify severe reactions in children at risk, how to administer emergency treatment, and how to seek for medical care. But not all patients respond to adrenaline or bronchodilators, and cases of death have been reported even after correctly using medication. Education on how to prevent reactions is, hence, at least as important as education on the management of acute reactions. Children at risk must be given a personal care plan informing on allergens, reactions to be expected, and how to treat them. The plan must be written in plain language, with clear steps on how to proceed. The plan and the medication must be stored in a safe and easy to access place. Education in a general basis can be provided in pre-grade and post-grade continued education activities for teachers. Collaboration from authorities would be needed for this. Specific activities can be organized; patient’s associations help is invaluable. Individual based education, with practical demonstration of use of medication, for patients, parents and school staff is crucial and can be life saving. Internet resources, available 24 hours a day, to provide and to reinforce education should not be forgotten: medical societies must not neglect this activity.

